# Does the Coronary Artery Bypass Grafting Impact the Survival of Men and Women Equally Compared to General Population? Results from KROK Registry and Polish Central Statistical Office

**DOI:** 10.3390/jcm13237440

**Published:** 2024-12-06

**Authors:** Grzegorz Hirnle, Adrian Stankiewicz, Maciej Mitrosz, Sleiman Sebastian Aboul-Hassan, Szymon Kocańda, Marek Deja, Jan Rogowski, Romuald Cichoń, Wojciech Pawliszak, Paweł Bugajski, Zdzisław Tobota, Bohdan Maruszewski, Piotr Knapik, Michał Krejca, Marek Cisowski, Tomasz Hrapkowicz

**Affiliations:** 1 Department of Cardiac Surgery, Medical University of Bialystok, 15-276 Białystok, Poland; stankiewiczad@wp.pl (A.S.); mitrosz@gmail.com (M.M.); skocanda@gmail.com (S.K.); 2 Department of Cardiac Surgery, Zbigniew Religa Heart Center “Medinet”, 67-100 Nowa Sol, Poland; s.aboul-hassan@inm.uz.zgora.pl (S.S.A.-H.); romuald.cichon@gmx.de (R.C.); 3 Department of Cardiac Surgery and Interventional Cardiology, Faculty of Medicine and Medical Sciences, University of Zielona Gora, 65-417 Zielona Gora, Poland; 4 Department of Cardiac Surgery, Upper-Silesian Medical Centre, Medical University of Silesia, 40-055 Katowice, Poland; mdeja@sum.edu.pl; 5 Department of Cardiac and Vascular Surgery, Medical University of Gdansk, 80-210 Gdańsk, Poland; janrog@gumed.edu.pl; 6 Department of Cardiac Surgery, Dr Antoni Jurasz Memorial University Hospital, 85-094 Bydgoszcz, Poland; pawliszakw@gmail.com; 7 Department of Cardiac Surgery, J. Struś Hospital, Poznań University of Medical Sciences, 61-701 Poznań, Poland; pawelbugajski@onet.eu; 8 Department of Pediatric Cardiothoracic Surgery, Children’s Memorial Health Institute, 04-730 Warszawa, Poland; ztobota@ecdb.pl.pl (Z.T.); bmar@ecdb.pl.pl (B.M.); 9 Department of Anesthesiology, Intensive Therapy and Emergency Medicine, Silesian Centre for Heart Diseases, Medical University of Silesia, 40-055 Zabrze, Poland; pknapik@sum.edu.pl; 10 Department of Cardiac Surgery, Medical University of Lodz, 90-419 Łódź, Poland; mkrejca@wp.pl; 11 Department of Cardiac Surgery, University Hospital, Institute of Medical Sciences, University of Opole, 45-040 Opole, Poland; marek.cisowski@usk.opole.pl; 12 Department of Cardiac Surgery, Vascular and Endovascular Surgery, and Heart Transplantology, Silesian Centre for Heart Diseases, Medical University of Silesia, 40-055 Zabrze, Poland; t.hrapkowicz@sccs.pl

**Keywords:** gender differences, coronary artery bypass grafting, long-term survival

## Abstract

**Objective:** The aim of this study was to evaluate the impact of coronary bypass surgery (CABG) on long-term mortality, comparing survival rates to those of the general population in Poland. **Methods:** The study was based on the Polish National Register of Cardiothoracic Surgical Procedures (KROK). Between January 2009 and December 2019, 133,973 patients underwent CABG. The study included all patients who underwent primary CABG. After excluding reoperations and patients with missing key data, there were 132,760 remaining patients who participated in the study. In order to compare patients who underwent CABG with the general population, data from Polish life expectancy tables from the Central Statistical Office (CSO) were used. **Results:** In the general population (GP), there is a consistent decrease in survival for both women and men throughout the entire observation period. The decline in survivability is more pronounced in the male group. Unlike the CABG group, which is at risk of perioperative mortality, there is no initial drop in survivability in the GP. The early mortality rate in CABG group within 30 days was significantly higher in the group of women than in men (3.51% compared to 2.19%, *p* < 0.001). The annual mortality rate was higher in the group of women (6.7% vs. 5.14%), and survival time was shorter (345.5 ± 0.4 vs. 351.2 ± 0.2 days, *p* < 0.001). However, the total mortality over a 13-year period of observation did not differ significantly between the groups (30.17% for women vs. 29.6% for men, *p* = 0.996) with survival time 10.08 ± 0.02 years in men vs. 10.06 ± 0.03 in women, *p* = 0.996. **Conclusions:** CABG surgery equalizes the probability of survival between genders. In long-term observation men have a greater survival benefit than women if compared to the predicted survival of the general population. These observations may provide a new perspective on the choice of revascularization strategy in relation to gender.

## 1. Introduction

Coronary artery bypass grafting (CABG) has established a strong position in the treatment of coronary artery disease, supported by observational and randomized studies. Numerous studies usually compare CABG with PCI, occasionally including comparisons between CABG and medical treatment [1,2,3,4]. However, contemporary data on the impact of CABG on long-term survival compared to the general population (GP) are very scarce [5,6].

Although CABG is responsible for only about 10% of all coronary revascularization procedures, given the prevalence of coronary artery disease (CAD) in modern society, it affects a substantial population [7]. Therefore, the continuous monitoring of revascularization procedure outcomes is necessary to select the appropriate patient population for surgical intervention.

The aim of this study was to assess the influence of CABG on long-term mortality following CABG and to compare survival rates with those of the GP in Poland.

## 2. Patients and Methods

This study utilized retrospective data collected and reported in accordance with the Polish National Registry of Cardiac Surgery Procedures: the KROK registry (https://krok.csioz.gov.pl/krok/;jsessionid=37F307A086DF526A7C53264C5382AA38?0, accessed on 1 September 2024) spanning from January 2009 to December 2019. The KROK registry encompasses all cardiac surgery procedures nationwide in Poland and is linked to the National Health Fund, which tracks all deaths in the country. The study was conducted according to the guidelines of the Declaration of Helsinki and was approved by the Institutional Review Board of the Medical University of Bialystok (APK.002.274.2024), Poland. All data were anonymized, and written, individual consent was not required. Early mortality was defined as death due to any cause within 30 days of surgery. Detailed information on database design and management has been published previously [8]. The current study was designed according to the Strengthening the Reporting of Cohort, Cross-Sectional, and Case-Control Studies in Surgery (STROCCS) guidelines [9].

To compare survival rates with the GP, the study simulated the survival time of individuals in Poland based on life expectancy tables from the Central Statistical Office (CSO) for the years 2009–2021 [10]. For each patient in the surgery database, the study simulated the survival time of an individual from the GP with the same sex and date of birth, estimating the probability of survival for day after surgery. The daily probability of survival was calculated assuming a constant risk throughout the year, following an exponential distribution, for which the cumulative probability of death is equal to that given in the life expectancy tables. The simulated data were used to determine the survival rates of individuals from the GP with demographic characteristics identical to those of the patients who underwent CABG.

### 2.1. Study Outcome

The outcome of the study is an assessment of the influence of CABG on long-term mortality assessed as all-cause mortality up to 13 years post-surgery and a comparison of survival rates with those of the general population in Poland.

### 2.2. Study Population

**Figure 1:**
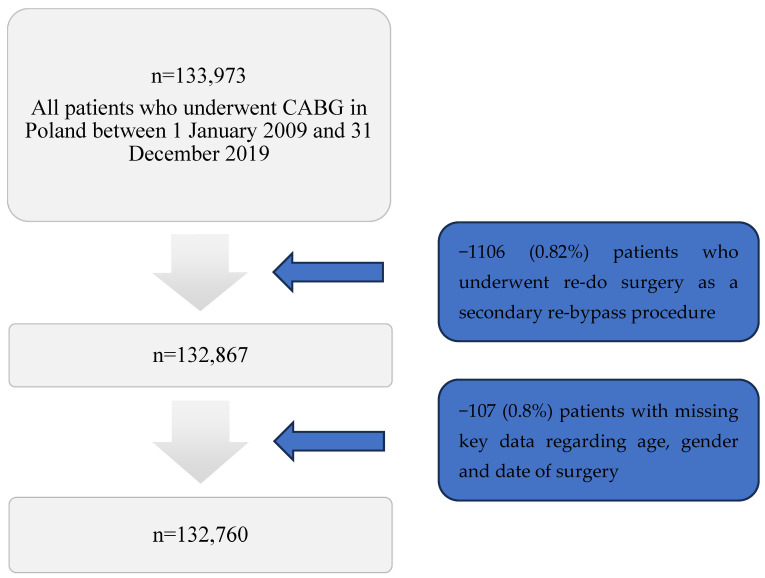
Patients flow-chart.

A total of 133,973 adult patients underwent isolated CABG in Poland during the 11-year study period (1 January 2009–31 December 2019) and were enrolled in the KROK Registry. The study included all patients who underwent primary, isolated CABG procedures. Patients who underwent re-do surgery as a secondary re-bypass procedure (1106 pts., 0.82%) and patients with missing key data regarding gender, age, and date of surgery (107 pts., 0.08%) were excluded from the study (Figure 1). Ultimately, the study group consisted of 132,760 patients, 99,998 males (75.3%) and 32,762 females (24.7%), compared with the same number from the GP. The selection and exclusion of specific patient groups aimed to ensure data consistency and accuracy in assessing long-term outcomes.

### 2.3. Statistical Analysis

To estimate life expectancy differences between CABG patients and the general population (GP), the study used a simulation model. It relied on life expectancy data from Poland’s Central Statistical Office, covering 2009–2021, with annual age- and gender-specific mortality risks for individuals up to 99 years old. For each patient in the surgical dataset, a simulated counterpart in the GP was matched by birth date and gender, beginning from the date of the patient’s surgery, to project life expectancy with comparable parameters across both groups.

To compare survival times between patients and matched simulated population data, a Cox proportional hazards model with a frailty (random) effect was applied to account for dependencies within matched pairs. The frailty effect captures unobserved heterogeneity within each pair, addressing correlations that arise from shared or unmeasured factors. This approach provides more accurate hazard ratio estimates by controlling for random influences specific to each pair, allowing for robust survival outcome comparisons between dependent groups.

Continuous variables were presented as mean and standard deviation (when non-parametric tests were used for comparison, median values were also used), while categorical variables were presented as absolute number percentages. Survival times were presented as mean and standard error. The t-Student, Mann–Whitney U, and Chi-squared tests were used to assess statistical significance where appropriate. Survival times were compared using logrank tests stratified by age to accommodate for age differences between sexes.

For analyses, a two-tailed *p*-value < 0.05 was considered statistically significant. The analyses and graphs were performed with the use of statistical software R version 4.2.1 2022 [11]. The matching procedure was carried out using the MatchIt R-package (version 4.6.0) [12]. Estimations of hazard functions were obtained with Muhaz R-package (version 1.2.6.4).

## 3. Results

The age and observation period are shown in Table 1.

The early, one-year, and entire follow-up mortality for all patients are shown in Table 2.

The curves depict a sharp decline in survival immediately after surgery in both groups, with a greater decline observed in women. The difference in favor of men within the first year after surgery is statistically significant (*p* < 0.001, HR 1.30, 95% CI 1.48–1.73). After 6 months, survival improves in both groups, with a more pronounced improvement in the women’s group, and after 4 years, the survival curve for women matches that of men. Subsequently, for most of the observation period, the curves overlap, indicating similar survival rates in both groups (*p* = 0.996). In the last year, a trend of improved survival is noted in the men’s group.

To compare survival times between patients and matched simulated population data, the Cox proportional hazards model with a frailty (random) effect was applied to account for dependencies within matched pairs. The frailty effect captures unobserved heterogeneity within each pair, addressing correlations that arise from shared or unmeasured factors. This approach provides more accurate hazard ratio estimates by controlling for random influences specific to each pair, allowing for robust survival outcome comparisons between dependent groups (Table 3).

Here we have two models, one for women and one for men. Each model includes two factors: group (patients or general population) and age. All effects are statistically significant, as indicated by low *p*-values, but the interpretation of hazard ratios (HR) is more insightful.

For both sexes, the age effect is similar: each year of age increases the mortality risk in women by 1.08 times (8%), and in men by 1.071 times (7.1%). The group effect is much larger, showing the difference between patients and the general population.

In women, HR = 1.414 means that female patients have a 1.414 times higher risk of death than women in the general population (41.4% higher). In men, this risk is smaller: male patients have a 1.093 times higher risk of death (9.3%).

**Figure 2:**
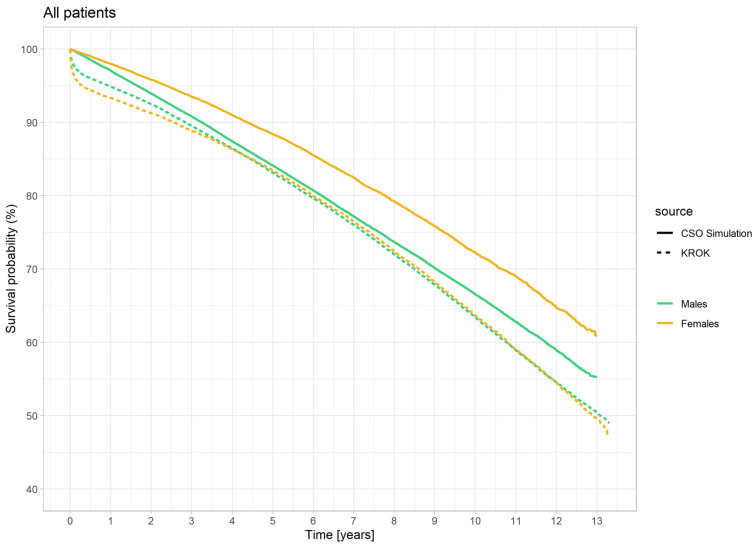
13-year survival probability curves of the simulated general population and survival curves of women (*n* = 32,762) and men (*n* = 99,998) in the CABG population. Data from Central Statistical Office and KROK Registry for the years 2009–2021.

To compare the survival of the CABG group and the GP, survival curves for women and men after CABG were overlaid with survival curves for the GP (Figure 2).

In GP survival curves, there is a consistent decrease in survival for both women and men throughout the entire observation period. The decline in survivability is more pronounced in the male group. Unlike the CABG group, which is at risk of perioperative mortality, there is no initial drop in survivability in the GP.

In the case of the females, after an initial 6-month decline in the survival of operated women, the curves level out and remain parallel for most of the observation period and never approaches the population curve.

Differently, after an initial 6-month decline in survival for operated men, the rate of survival decline in operated men becomes slower than the population curve. Around the 3rd year after surgery, it approaches the population curve, and for the next 5 years, the curves run almost parallel. After 9 years post-operation, the survival curve for operated men gradually begins to decline more steeply than the population curve.

**Figure 3:**
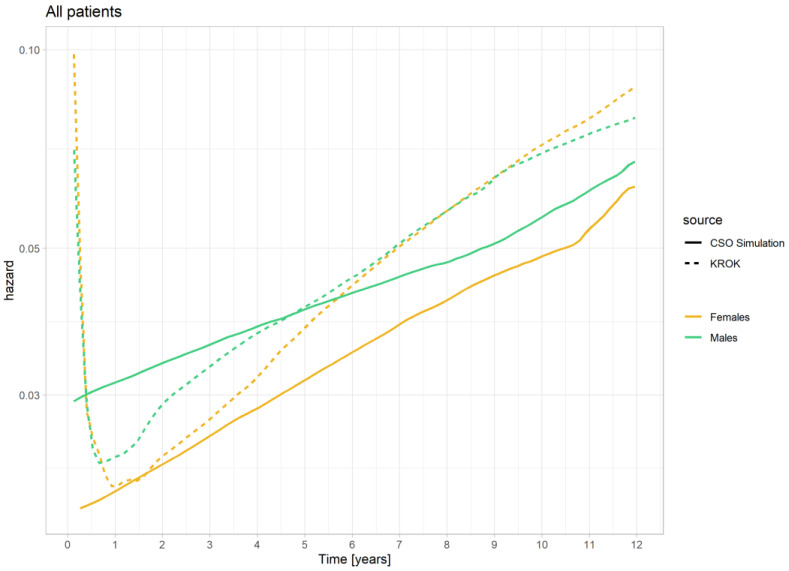
Death hazard within a specified period for females (*n* = 32,762) and males (*n* = 99,998) after CABG surgery and the general Polish population.

The above-mentioned relationships are more clearly presented not as survival probability but as death hazard, as presented in Figure 3.

### 3.1. Standardized Mortality Ratios

Standardized Mortality Ratios (SMR) values greater than 1 indicate higher mortality in the CABG group. For example, an SMR of 29.07 within 30 days post-surgery in women under 70 years of age suggests that the mortality rate in women who underwent CABG is nearly 30 times higher than that of the general population. SMR values less than 1 indicate lower mortality in the CABG group. For instance, an SMR of 0.92 between 7 and 12 months post-surgery in men under 70 years of age means that men who underwent CABG have an 8% lower mortality rate compared to the general population.

**Figure 4:**
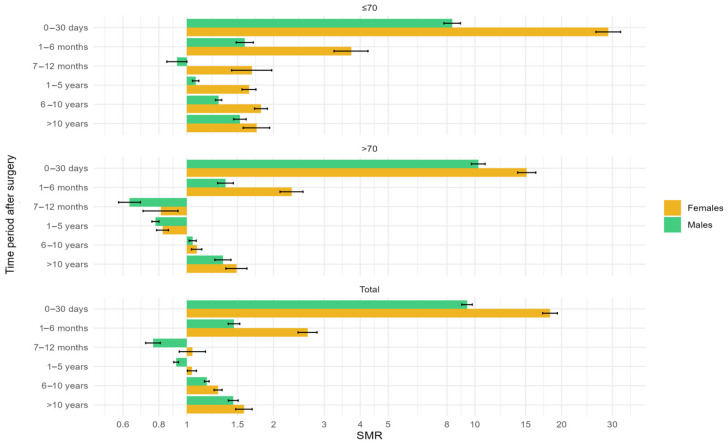
Standardized Mortality Ratios (SMR) by gender, age group, and time after surgery.

Confidence intervals (C.I.) shown as whiskers on the graphs allow for the assessment of statistically significant differences (Figure 4). For example, in the overall group (without age stratification), 1–5 years post-surgery, the SMR for men is 0.92 with a confidence interval of 0.9–0.94, which means that men who underwent CABG have a lower risk of mortality compared to the general population. In contrast, the SMR for women in the same period is 1.04 with a confidence interval of 1.00–1.04, indicating that women have a higher mortality rate than the general population. Additionally, since the confidence intervals do not overlap (0.9–0.94 for men and 1.00–1.04 for women), we can conclude that there is a statistically significant difference between the SMRs for women and men 1–5 years after surgery.

Confidence intervals can also be used to determine whether the SMR is significantly above or below 1 (i.e., whether mortality in the CABG group significantly exceeds or is lower than that of the general population). If the confidence interval includes 1 (e.g., for women in the total group between 7–12 months post-surgery: 0.94–1.16), it indicates that the SMR is not significantly different from 1, meaning we cannot conclude whether the difference is positive or negative, i.e., no statistically significant difference is observed. If the confidence interval does not include 1 (and both bounds of the interval are either below or above 1), it means the SMR is statistically significant, indicating a meaningful difference between the CABG group and the general population.

The above-mentioned relationships are more clearly presented in the Table 4 and Figure 4.

### 3.2. Risk of Death

The observation of the risk of death was terminated after year 12 because estimating the death hazard over a longer follow-up period is unstable and unreliable due to the small number of follow-up cases and deaths.

If we compare operated women and men, we can observe that immediately after surgery, the woman’s curve is higher, indicating a higher risk of death. Then, for 8 months after surgery, both curves overlap, signifying a similar risk of death for both women and men. The very high postoperative risk of death quickly decreases, and around the 8-month mark, the negative perioperative effect disappears, resulting in the lowest risk of death throughout the observation period. For women, this process takes longer, and the risk continues to decrease even up to 1 year, eventually reaching a lower level than that of men one year after surgery. From this point onwards, the risk of death in both groups increases, with women catching up to men’s risk 6.5 years after surgery. After this period, the risk of death becomes identical in both groups for the next 3 years (the curves overlap). After a decade of follow-up, the mortality risk for men begins to fall below that for women.

In the GP, there is no initial surge in the risk of death as an effect of surgery. From the beginning, the risk of death in men is noticeably higher than in women. Then, throughout the entire observation period, there is a gradual increase in the risk of death, with a greater increase in women (the curves come closer together). However, the risk of death in men surpasses that of women throughout the entire observation period.

Comparing the risk of death in operated patients to the GP, categorized by gender, we can see that operative risk for women is higher but the decrease in perioperative risk is greater for men. However, unlike men, the risk of death in women never falls below the population risk for their gender. During the period between the first and second year after surgery, the risk of death for operated women is similar to the population risk for women. After this period, the risk of death for operated women continues to rise steadily compared to the GP of women.

In the case of operated men, immediately after the procedure, the risk of death, similar to women, is much higher than in the GP. However, it quickly decreases, and even before the 6-month mark, the curves intersect. From that point on, the risk of death for operated men is lower than that of men in the GP. Subsequently, in both groups, the risk increases with age, but it does so more rapidly in operated men. 4.5 years after surgery, the risk curves for death intersect again, and operated patients once again have a higher risk of death than men in the GP. From that moment to 9 years after surgery, the risk of death for operated men increases more rapidly than in the GP, and then it remains parallel in both groups until the end of the observation period.

## 4. Discussion

Our observation showed an improvement in survival after CABG compared to the GP, particularly prominent in the male group, where, in certain time intervals, survival is better than in the GP.

The survival of CABG patients was compared with a simulated GP. Between 2009 and 2019, a total of 1,377,885 coronary revascularization procedures were performed, with percutaneous procedures (PCI) accounting for 1,243,912 (89.2%) and 133,973 CABG accounting for 10.8% [7]. Both revascularization methods exhibited a declining trend while maintaining a similar percentage relationship throughout the observation period [7]. The average number of CABG surgeries was 314/1,000,000 inhabitants, with predominance among males (488.8/1,000,000) compared to females (150.1/1,000,000).

CABG surgery is perceived as a highly effective procedure in terms of long-term outcomes but is associated with significant invasiveness and notable surgical risk [1,4,5,6,13,14]. However, there is very limited data comparing survival after CABG and the GP [5,15]. Two non-modifiable factors have the most significant impact on mortality: the age and gender of patients. Both are considered in all available surgical risk scales and can be obtained from life tables of the GP and compared with the population of patients undergoing CABG. We decided to assess the impact of CABG of both female and male patients on long-term survival and compare it with the survival of GP.

These observations may have significance regarding the current reduction in AHA recommendations for CABG in patients with stable angina with EF > 35% from Class I to Class IIa. Mehafney et al. notes that this change was based on one study, which was not designed to assess survival after CABG but rather to compare outcomes between PCI and OMT. The inclusion of CABG in this study was incidental. The CABG group was included in the PCI group and accounted for just under 8% of the study population. Mehafney’s latest large registry, comparing PCI and CABG in terms of survival and other endpoints, demonstrated a significant advantage of CABG over PCI. This implied a flawed assumption of equivalence between the two methods [16].

### 4.1. Probability of Survival

The only contemporary multicenter registry comparing a large population of postoperative patients with the GP is the study published in 2017. The authors demonstrated significantly increased early and late mortality in the CABG group compared to average Danes but also showed a trend of improving CABG outcomes over the last three decades [5].

A pioneering study in this field is the Dutch study involving 1071 patients operated on between 1971 and 1980, with follow-up of 20-years, later exended to 30-years [17,18]. The study did not show differences in survival after surgery between genders but demonstrated lower survival rates in the CABG group compared to the GP over the entire 20-year observation period. However, after an additional 10 years of follow-up, survival rates became equal between the groups, with the CABG group at 16% and the GP at 19%. This study is particularly valuable as it covers practically the entire lifespan of the evaluated population. The convergence of survival between both groups in the very distant observation suggests the high value of CABG in the treatment of CAD. Other studies showed comparable results regarding survival in the operated population and the GP, but the observation period was significantly shorter [19,20].

The most recent study assessed long-term survival in the CABG group compared to the general Norwegian population including a group of 4044 patients, of which 17.7% were women [15]. The authors found increased early and mid-term mortality and better long-term survival among women [15]. This is the only publication consistent with our observations. The similarity between these studies lies in the fact that both included the European population and covered a similar period of operations, but they greatly differ in the size of the study groups, as the Norwegian study was a single-center study, while ours was a nationwide registry.

In our study, immediately after CABG, both women and men experienced a sharp decline in survival, with a greater decline in women, associated with the risk of the surgical procedure itself. Three months after the procedure, the decline in survival decreased in both groups, with more pronounced improvement in women, and after about 4 years, the survival curve for women caught up and overlapped with the curve for men. Throughout the rest of the observation period, survival was similar in both groups (the curves overlapped for most of the observation period). Therefore, despite the increased surgical risk in women, the long-term impact of CABG on survival appears to be the same for men. This means that considering the higher overall mortality rate in men in the GP, CABG levels the playing field for long-term survival between men and women.

Male mortality exceeded female mortality and increased throughout the observation period. This probably means that although the increased surgical risk in women provides a survival advantage for men in the early postoperative period, men have a shorter lifespan in the long-term observation, which is likely related to the global trend of a higher mortality rate among men [20].

In our population, women were over 3 years older than men at the time of operation. However, it seems that in distant observation, this factor was offset and the phenomenon of male overmortality began to predominate [3,20], as evidenced in the survival curves of the GP.

### 4.2. Probability of Death

Among operated patients, the risk of death for women is initially higher or the same as for men at the beginning and end of the observation period. Between 1 and 6 years after surgery, the risk for women is higher. This means that CABG evens out and, in some segments of the observation period, reduces the risk of death for the male population compared to the female population.

Patients with CAD are burdened with atherosclerosis and its complications, as well as risk factors for CAD such as hyperlipidemia, hypertension, and smoking. Moreover, the most common cause of death in the GP is cardiovascular disease. Additionally, patients in the CABG group undergo an assessment for other conditions that may affect prognosis, such as cancer, during the qualification process, which disqualifies patients from CABG. In the GP, there is obviously no such selection.

In summary, the separately presented results of survival after CABG and the GP are expected and obvious. The probability of survival and the risk of death for operated men and women obtained in our study confirm the results obtained in some previous studies, even more clearly, given the large sample size. Similarly, the survival results for the GP obtained in our study represent the expected outcome due to the well-known male overmortality. Only the overlay of survival curves for patients after CABG and the GP, divided by gender, yielded surprising and unexpected results.

The main limitation of this analysis is its retrospective nature based on registry data. Over the last 10 years, the characteristics of operated patients, their age and pharmacological treatment have changed. Improved surgical techniques, including the more frequent total arterial revascularization and procedures without cardiopulmonary bypass, have improved long-term survival after CABG surgery over the years. The survival of the GP is also changing, with predicted survival lower 10 years ago than today. This may cause inaccuracies in the proposed analysis, but we believe that the large group of patients included in this study significantly reduces these discrepancies. Interpreting the results of both our study and other registry-based studies evaluating mortality in patients operated on for CAD compared to the GP should be considered factors that cannot be eliminated in registry studies and may influence the obtained results.

## 5. Conclusions

The results of our study suggest that coronary revascularization strategies may benefit from reconsideration and adaptation based on patient sex. The findings indicate that men particularly benefit from surgical revascularization, as CABG appears to align the survival rates of male patients more closely with that of the general male population. This implies that tailoring revascularization approaches according to sex-specific outcomes could enhance the effectiveness of treatments and long-term survival, especially in male patients. However, a similar effect of CABG was not observed among women, indicating the need to consider alternative treatment strategies for this group.

## Figures and Tables

**Table 1:** The age and duration of observation of CABG group.

		All Patrients (*n* = 132,760)				
		N	Median	IQR (Q1–Q3)		*p*
Age	Women	32,762	69.01	62.90	74.85	<0.001
	Men	99,998	64.81	59.08	71.46	
	All	132,760	65.85	59.85	72.53	
Follow-up Time (years)	Women	32,762	6.97	4.17	9.71	<0.001
	Men	99,998	6.75	4.05	9.57	
	All	132,760	6.81	4.08	9.60	
*p*-Mann-Whitney test						

*p*-log-rank test stratified by age.

**Table 2:** The mortality of patients after CABG.

Time Period (Days)	All Patients (*n* = 132,760)						
	Men (*n* = 99,998)		Women (*n* = 32,762)		*p*	HR	95% CI
	Deaths N (%)	Mean Survival Time ± SE	Deaths N (%)	Mean Survival Time ± SE			
0–30	2188 (2.19%)	29.54 ± 0.01	1149 (3.51%)	29.25 ± 0.02	<0.001	1.60	(1.48–1.73)
0–183	4011 (4.02%)	177.47 ± 0.09	1832 (5.60%)	174.77 ± 0.20	<0.001	1.39	(1.31–1.48)
0–365	5138 (5.14%)	351.15 ± 0.20	2191 (6.70%)	345.51 ± 0.43	<0.01	1.30	(1.23–1.37)
Entire follow-up	29,601 (29.60%)	2489.19 ± 3.48 (6.79 years)	9883 (30.17%)	2481.08 ± 3.98 (6.88 years)	0.001	1.02	(1.00–1.04)

*p*-log-rank test stratified by age.

**Table 3:** Survival times comparison between patients and the matched simulated population.

Fixed Effects	Women		Men	
	HR	*p*	HR	*p*
Patient (vs. population)	1.414	<0.001	1.093	<0.001
Age	1.080	<0.001	1.071	<0.001

**Table 4:** Standardized Mortality Ratios (SMR) by gender, age group, and time after surgery.

Time After Surgery	Gender	Age Group								
		≤70			>70			Total		
		Number of Deaths		SMR (95% CI)	Number of Deaths		SMR (95% CI)	Number of Deaths		SMR (95% CI)
		CABG Group	Simulated Population Data		CABG Group	Simulated Population Data		CABG Group	Simulated Population Data	
0–30 days	Females	407	14	29.07 (26.32–32.04)	742	49	15.14 (14.07–16.27)	1149	63	18.24 (17.20–19.32)
0–30 days	Males	893	107	8.35 (7.81–8.91)	1295	126	10.28 (9.73–10.85)	2188	233	9.39 (9.00–9.79)
1–6 months	Females	216	58	3.72 (3.24–4.26)	467	202	2.31 (2.11–2.53)	683	260	2.63 (2.43–2.83)
1–6 months	Males	835	525	1.59 (1.48–1.70)	988	725	1.36 (1.28–1.45)	1823	1250	1.46 (1.39–1.53)
7–12 months	Females	155	92	1.68 (1.43–1.97)	204	251	0.81 (0.71–0.93)	359	343	1.05 (0.94–1.16)
7–12 months	Males	611	661	0.92 (0.85–1.00)	516	815	0.63 (0.58–0.69)	1127	1476	0.76 (0.72–0.81)
1–5 years	Females	1258	765	1.64 (1.55–1.74)	1745	2118	0.82 (0.79–0.86)	3003	2883	1.04 (1.00–1.08)
1–5 years	Males	6011	5598	1.07 (1.05–1.10)	4829	6204	0.78 (0.76–0.80)	10,840	11,802	0.92 (0.90–0.94)
6–10 years	Females	1480	818	1.81 (1.72–1.90)	2304	2129	1.08 (1.04–1.13)	3784	2947	1.28 (1.24–1.33)
6–10 years	Males	6305	4889	1.29 (1.26–1.32)	4833	4609	1.05 (1.02–1.08)	11,138	9498	1.17 (1.15–1.19)
>10 years	Females	353	202	1.75 (1.57–1.94)	552	371	1.49 (1.37–1.62)	905	573	1.58 (1.48–1.69)
>10 years	Males	1552	1015	1.53 (1.45–1.61)	933	699	1.33 (1.25–1.42)	2485	1714	1.45 (1.39–1.51)

## Data Availability

The raw data supporting the conclusions of this article will be made available by the authors on reasonable request.
